# Intracellular ion concentrations and cation-dependent remodelling of bacterial MreB assemblies

**DOI:** 10.1038/s41598-020-68960-w

**Published:** 2020-07-20

**Authors:** Dávid Szatmári, Péter Sárkány, Béla Kocsis, Tamás Nagy, Attila Miseta, Szilvia Barkó, Beáta Longauer, Robert C. Robinson, Miklós Nyitrai

**Affiliations:** 1 Department of Biophysics, Medical School, University of Pécs, Pécs, Hungary; 2 MTA-PTE Nuclear-Mitochondrial Interactions Research Group, Pécs, Hungary; 3 Department of Medical Microbiology and Immunology, Medical School, University of Pécs, Pécs, Hungary; 4 Department of Laboratory Medicine, Medical School, University of Pécs, Pécs, Hungary; 5 Szentágothai Research Center, University of Pécs, Pécs, Hungary; 6 School of Biomolecular Science and Engineering (BSE), Vidyasirimedhi Institute of Science and Technology (VISTEC), Rayong, Thailand; 7 Research Institute for Interdisciplinary Science (RIIS), University of Okayama, Okayama, Japan

**Keywords:** Biophysics, Microbiology

## Abstract

Here, we measured the concentrations of several ions in cultivated Gram-negative and Gram-positive bacteria, and analyzed their effects on polymer formation by the actin homologue MreB. We measured potassium, sodium, chloride, calcium and magnesium ion concentrations in *Leptospira interrogans*, *Bacillus subtilis* and *Escherichia coli*. Intracellular ionic strength contributed from these ions varied within the 130–273 mM range. The intracellular sodium ion concentration range was between 122 and 296 mM and the potassium ion concentration range was 5 and 38 mM. However, the levels were significantly influenced by extracellular ion levels. *L. interrogans*, *Rickettsia rickettsii* and *E. coli* MreBs were heterologously expressed and purified from *E. coli* using a novel filtration method to prepare MreB polymers. The structures and stability of Alexa-488 labeled MreB polymers, under varying ionic strength conditions, were investigated by confocal microscopy and MreB polymerization rates were assessed by measuring light scattering. MreB polymerization was fastest in the presence of monovalent cations in the 200–300 mM range. MreB filaments showed high stability in this concentration range and formed large assemblies of tape-like bundles that transformed to extensive sheets at higher ionic strengths. Changing the calcium concentration from 0.2 to 0 mM and then to 2 mM initialized rapid remodelling of MreB polymers.

## Introduction

In bacterial cultures, the survivability of cells is influenced by extracellular salt effects, and cells adapt to changes in osmolarity by changing their intracellular ionic strength^1^. During the process of osmoadaptation, bacteria adjust to variant osmotic conditions by sodium/potassium uptake and release from and to the surrounding medium^2^. Many non-halophilic bacteria show a two-phase adaptation reaction to hyperosmotic shock^3^. In the first phase, the cells take up a large amount of K^+^ via specific ion transport systems. This step is necessary to compensate for the efflux of water that occurs due to the increase in the exterior osmolality^4^. High intracellular concentrations of K^+^ can have negative effects on protein function, protein binding to DNA and protein synthesis^5,6^. The second phase frequently involves the synthesis or uptake of compatible solutes^7,8^ and the efflux of K^+^^3,4^. The osmotic shock response of *E. coli* causes an increase of intracellular Na^+^ and K^+^ concentrations up to several hundred millimolar^9^. In *B. subtilis*, the recovery of turgor after moderate osmotic change, by the addition of 0.4 M NaCl, increased intracellular K^+^ levels from a basal value of 350 mM to 650 mM within 1 h^10^.

Bacterial cell shape is determined by the structure of the cell wall. The cell wall is a rigid crosslinked meshwork, the shape of which is maintained by the cellular turgor pressure. Bacteria can be subdivided into Gram-negative species that have a thin peptidoglycan layer and both an inner cytoplasmic and outer membrane, and Gram-positive species with only one cytoplasmic membrane and a thick peptidoglycan layer comprised largely of teichoic acids. Both types of bacteria use MreBs to organize peptidoglycan and teichoic acid synthesizing enzymes required for cell-wall assembly, growth and function^11^. The supramolecular morphology of MreB is known to be complex. Initial immunofluorescence microscopic studies with MreB fused to fluorescent proteins^12,13^ indicated that MreB forms spiral-like patterns under the cell surface. Results from super-resolution microscopy techniques increased the level of detail, demonstrating that MreB forms shorter helical segments, seen as patches, that associate with the inner membrane of the bacteria^14,15^.

Important observations were made in three independent studies showing the coupling of the dynamics of MreB to cell-wall synthesis in two evolutionary different species; Gram-negative *Escherichia coli*^16^ and Gram-positive *Bacillus subtilis*^14,15^. These results established that MreB polymers rotate around the long axis of the cell on a time scale of minutes. Although MreB functions had previously been related to its polymerization, these studies showed that the rotational motion was dependent on cell-wall synthesis activity. If cell-wall precursors were depleted, or cell-wall synthesis enzymes were inhibited with antibiotic drugs, MreB rotation stopped. MreB appears to coordinate the activity of enzymes involved in bacterial growth^17,18^. *E. coli* rapidly restores its cytoplasmic volume after osmotic shock^19^, and short polymers of MreB are required for the recovery of the rod shape^20^. A minimal degree of rotational motion by MreB polymers is needed to maintain cell shape, however this seems to be independent of the intracellular turgor pressure^21^. MreB is linked to the cell wall via RodZ^20^ in order to manage peptidoglycan insertion. Cell recovery after osmotic shock initially proceeds at a slow growth rate until the inner membrane, with bound MreB, can reattach to the cell wall, whereupon normal growth rates are restored^22^. Furthermore, inhibition of MreB polymerization with the drug A22 led to the loss of cell shape^23^, indicating an interdependence of MreB polymerization and cell wall synthesis. Kawei and colleagues used a co-immunoprecipitation assay combined with mass spectroscopy to identify proteins that interact with either of the three *B. subtilis* MreB isoforms; MreB, Mbl, and MreBH. More than 100 potential MreB-interacting proteins were identified. They concluded that MreB may provide a scaffold for cell-wall synthesis enzymes and cytoplasmic enzymes required for building cell-wall precursors. By contrast, *Escherichia coli* cells can grow in the complete absence of MreB^24^, suggesting that while MreB may enhance the efficiency of cell wall synthesis, it is not essential for the process. A possible alternative interpretation is that MreB spatially determines or constrains the potential sites of cell-wall synthesis, as suggested by Furchtgott et al.^25^ and by van Teeffelen et al.^16^. In silico modelling of a growing cell wall showed that helical and stiff arrangements of MreB polymers may stabilize the rod-like cell shape in Gram-negative bacteria by enabling a uniform density of peptidoglycan insertion events in the cell wall. This mechanism assumes stiff MreB filaments that bridge the spatial gaps in the cell wall^26^. In vitro high salt conditions can cause MreB polymers to stack into stable sheets^27^. Such morphologies may be related to the in vivo functions, as MreB structures arranged in helical patterns are required for inner membrane and cell shape stability^28^. However, the roles of the inherent dynamic properties, remodelling and reorganization of MreB in the cell wall maintenance and cell division still need to be clarified.

In vitro solution conditions have significant effects on the organization and function of MreB. Intracellular ion levels can prevent MreB’s spontaneous assembly while allowing for site-specific assembly of short polymers^27,29,30^. MreB polymerization was also shown to be sensitive to the type of bound nucleotide^31^. Polymerization of MreB from *Thermotoga maritima* (*Tm*-MreB) can be initiated by the addition of ATP. The rate of polymerization accelerates with increasing MgCl_2_ concentration^32^, while CaCl_2_ does not have a marked influence on the process. However, calcium influx plays an important role in cell cycle and chemotaxis of *E. coli* cells^33^. Several important questions remain regarding the roles of ions in the structure and function of MreB filaments. Which ions are essential for the stability for MreB polymers, what conditions can mobilize the MreB cytoskeleton, and how the balance between assembly and disassembly is regulated by ionic changes, all require further investigation.

Due to the characteristic helical morphology of the *Leptospira interrogans* cells, which is remarkably distinct from rod-shaped bacteria, these cells provide an exciting platform to understand the molecular basis of the organization and function of MreB polymers^34^. The phylum *Spirochaetes* comprises organisms that have distinct cellular morphologies ranging from helical to flat wave-shaped cells. *Leptospira interrogans* has a long (~ 10 μm) but thin (~ 0.2 μm) helical cell shape and its motility is driven by the rotation of two periplasmic flagella. These bacteria use the flagella to create corkscrew motility. Currently it is not known whether the formation of the cell wall in these cells relies on a different mechanism of their peptidoglycan synthesis. Also, only limited information is available about the set of proteins participating in the process, though the MreBs of *Leptospira interrogans* is likely to have a role in controlling cell morphology^35^.

Here we have analyzed the intracellular ionic conditions in *Escherichia coli* and *Leptospira interrogans* as examples Gram-negative bacteria species, and *Bacillus subtilis* as a Gram-positive bacteria species. We have characterized the polymerization of MreBs from different Gram-negative species, *Leptospira interrogans* MreB (*Li*-MreB) and *Rickettsia rickettsii* MreB (*Rr-*MreB) in vitro, under various ionic conditions. We compared the properties of *Li*-MreB and *Rr-*MreB polymers to *Escherichia coli* MreB (*Ec*-MreB) filaments as a reference. Based on these data, we propose a model for the regulation of MreB assembly and remodelling by ion flux.

## Results

### Intracellular ion concentrations in bacteria

**Figure 1:**
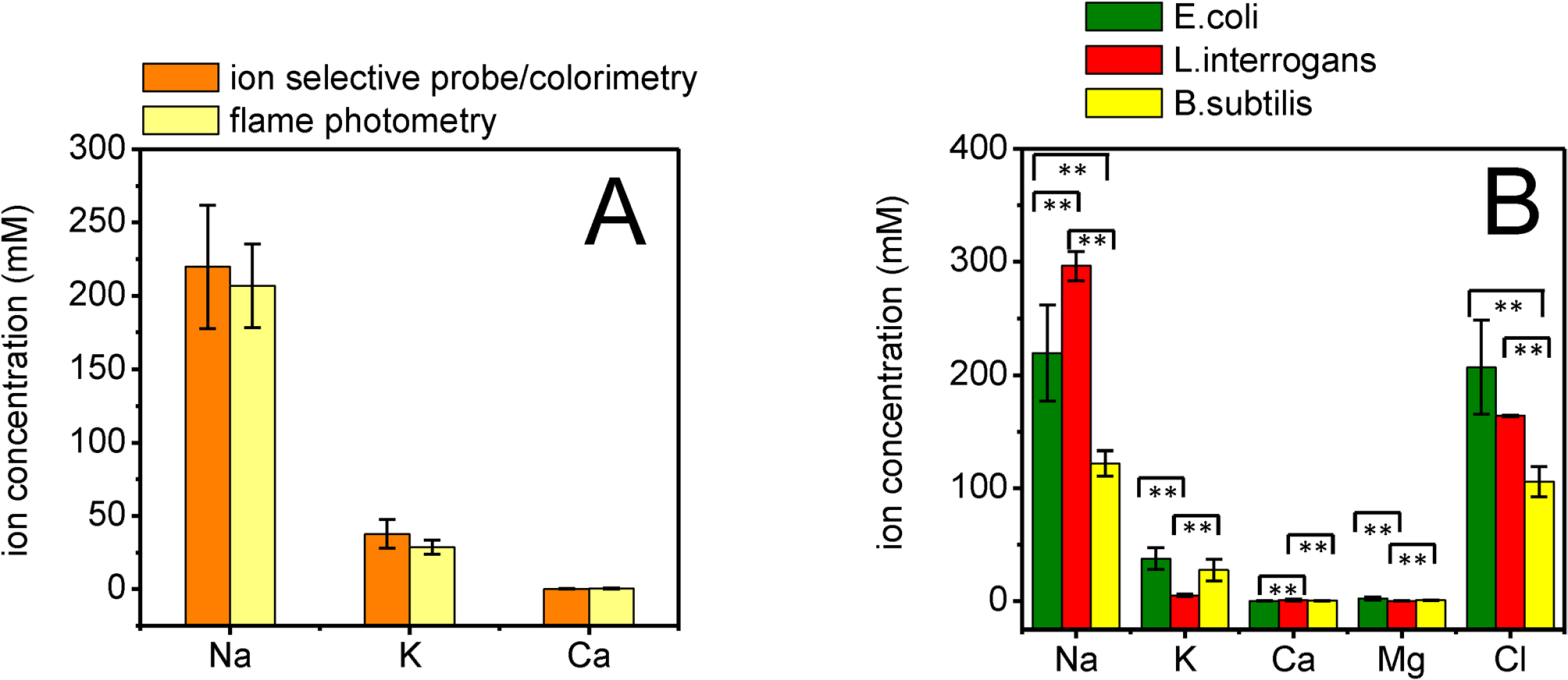
Intracellular ion concentrations in bacteria. (**A**) Comparison of the intracellular ion concentrations of *Escherichia coli* measured with ion-selective electrode/colorimetry (orange columns) and flame photometry (yellow columns). Error bars refer to mean ± SD of three independent measurements. There is no statistically significant difference between the data from the two methods, *p* > 0.05. (**B**) The data obtained using ion-selective electrode/colorimetry to determine the internal ion concentrations of different species. Gram-negative bacteria *Leptospira interrogans* (red columns) and *Escherichia coli* (green columns), Gram-positive *Bacillus subtilis* (yellow columns). Error bars refer to mean ± SD of six independent measurements. Horizontal bars with two asterisks indicate statistical significances between two values, based on a Students *t*-test, *p* < 0.005.

Ion-selective electrode/colorimetry is able to measure concentrations of free ions, while flame photometry measures the total concentrations of ions, including ions bound to proteins and DNA. To compare these two methods, we measured the intracellular concentration of Na^+^, K^+^ and Ca^2+^ in samples extracted from *Escherichia coli* using both ion-selective electrode/colorimetry and flame photometry. The two methods detected similar concentrations of the three ions (Fig. 1A) (with no statistically significant difference, *p* > 0.05), indicating that the ion extraction method used here was effective in releasing the majority of bound ions. Ion-selective electrode/colorimetry is able to measure more types of ions than flame photometry, therefore in subsequent experiments only ion-selective electrode/colorimetry was used to measure ion concentrations (including Na^+^, K^+^, Ca^2+^, Mg^2+^ and Cl^−^). Our aim was to understand whether a relationship exists between the intracellular ionic conditions and the cell shapes of two Gram-negative species, *Leptospira interrogans* (*Li*), which is a *Spirochaete* with a helical shape, and *Escherichia coli* (*Ec*) with rod-shaped cell morphology*.* For comparison, we examined a Gram-positive species *Bacillus subtilis* (*Bs*), which also has a rod-shaped cell. The ion concentrations determined in all three species are presented in Fig. 1B*.* Sodium ion concentrations were high in all three species: 296 ± 12 mM in *Li*, 219 ± 42 mM in *Ec* and 122 ± 11 mM in *Bs*, and show statistically significant differences. By contrast, potassium ion concentrations were relatively low: 38 ± 10 mM and 27 ± 10 mM in *Ec* and *Bs*, respectively, and significantly lower 5 ± 1 mM in *Li*. The calcium ion concentration was 1 ± 0.08 mM (significantly higher than for *Ec* and *Bs*) and the magnesium concentration was 0.38 ± 0.2 mM in *Li* (statistically significantly lower than for the other two species). Interestingly, in *Ec* and *Bs* the calcium ion concentrations were sub-millimolar, while the magnesium ion concentration was between 1 and 2 mM. Chloride ions are known to be the major anion composition of bacterial cells. Here, we measured the chloride ion concentrations to be 164 ± 0.7, 207 ± 41 and 106 ± 13 mM in *Li*, *Ec* and *Bs*, respectively (there is a statistically significant difference only between *Li* and *Ec*). Na^+^, K^+^, Cl^−^, Ca^2+^, and Mg^2+^ are the major contributors to intracellular ionic strength^36–38^. Calculation (Eq. 1) of the intracellular ionic strengths in the differences species, based on these ions, gave values of 235 mM (*Li*), 237 mM (*Ec*) and 130 mM (*Bs*). Despite of the different levels between species, the ratios between various ions in a single species were similar between the three species.

### Polymerization of MreB under various salt conditions

**Figure 2:**
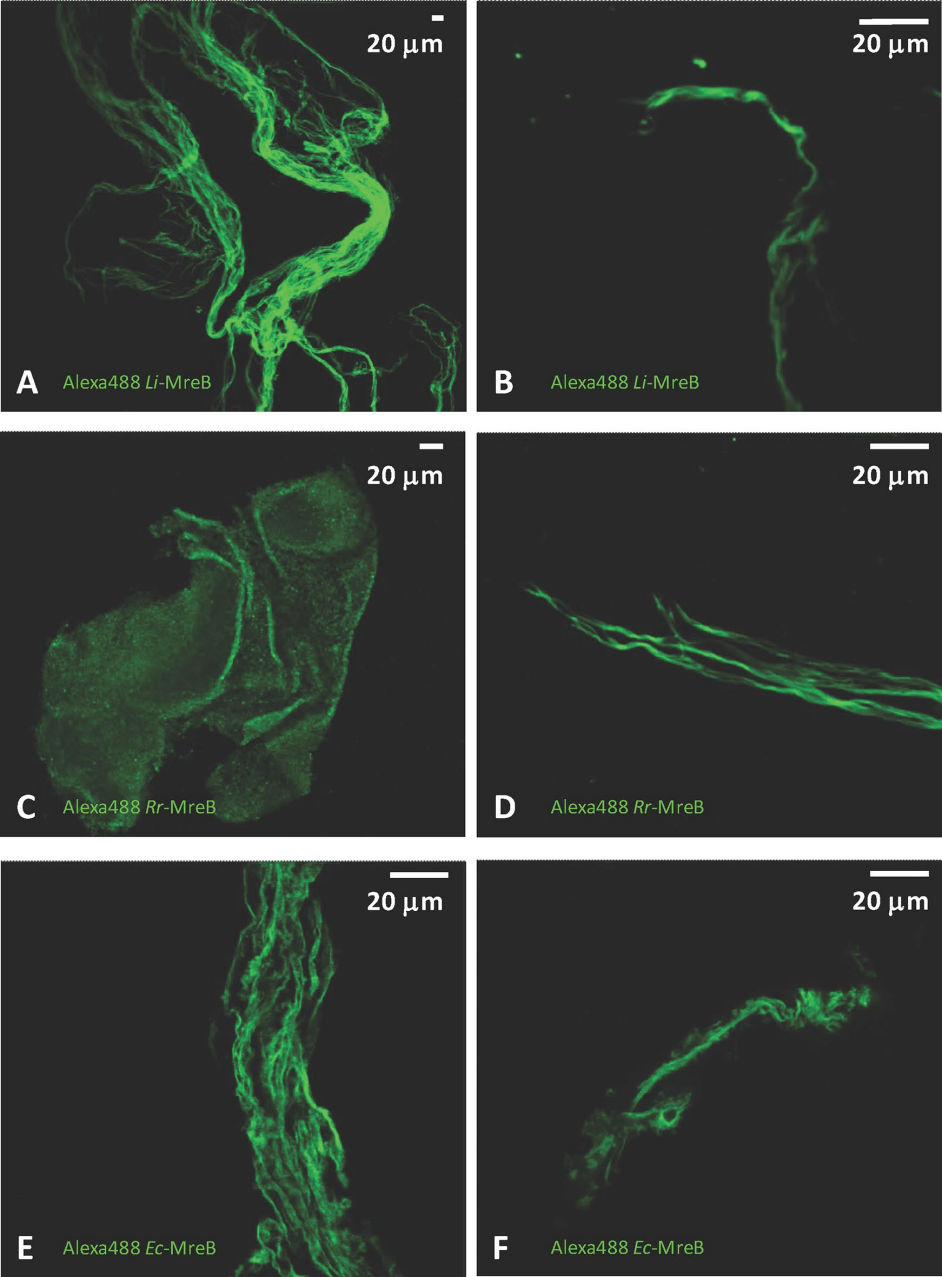
Microscopy imaging of MreB polymers isolated under different ion conditions. (**A** and **B**) PF filtered, Alexa488-labeled *Li*-MreB polymers form large superstructures (**A**) in polymerizing ionic conditions (300 mM KCl, 2 mM MgCl_2_, 0.1 mM CaCl_2_) (**B**) in its intracellular ionic conditions determined in this study (296 mM NaCl, 5 mM KCl, 0.38 mM MgCl_2_, 0.99 mM CaCl_2_). (**C** and **D**) PF filtered, Alexa488-labeled *Rr-*MreB polymers form sheets or assemblies in an ionic strength dependent manner (**C**) under high ionic conditions (300 mM KCl, 2 mM MgCl_2_, 0.1 mM CaCl_2_), (**D**) and under lower KCl conditions (200 mM KCl, 2 mM MgCl_2_, 0.1 mM CaCl_2_). (**E** and **F**) PF filtered, Alexa488-labeled Ec-MreB polymers form large superstructures (**E**) under high ionic conditions (300 mM KCl, 2 mM MgCl_2_, 0.1 mM CaCl_2_) (Ec-MreB polymers form similar assemblies to Li-MreB), (**F**) in its intracellular ionic conditions determined in this study (219 mM NaCl, 38 mM KCl, 2.38 mM MgCl_2_, 0.24 mM CaCl_2_).

To assess the impact of the different ion conditions on MreB polymer formation, we overexpressed MreBs from three Gram-negative species [*E. coli*, *Leptospira interrogans* and *Rickettsia rickettsii* (*Rr*)] in *E. coli*. The MreBs were subsequently purified with a novel polymer filtration method (PF) and a previously published denaturation method^39^. In the PF method MreBs were polymerized under high salt conditions (50–300 mM KCl, 2 mM MgCl_2_, 0.1 mM CaCl_2_) and then filtered (Supp. 1A,B,C). In a previous study, the filament-forming properties of *Li*-MreB were characterized by following polymerization through measuring the increase in light scattering on polymerization^39^. The optimal salt condition for *Li*-MreB polymerization was determined to be 300 mM KCl, 2 mM MgCl_2_ and 0.1 mM CaCl_2_. However, in our present study we determined that *Rr-*MreB required only 200 mM KCl for optimal polymerization (Supp. 2B,C,D) in the presence of MgCl_2_ (2 mM) and CaCl_2_ (0.1 mM). In the absence of magnesium ions, *Rr-*MreB shows no increase in light scattering indicative of the absence of polymerization, similar to the properties observed for *Li*-MreB (Supp. 2E)^39^. In the absence of potassium ions, the light scattering level was elevated in response to the addition of millimolar concentrations of divalent cations, which was caused by the precipitation of *Rr-*MreB (Supp. 2E). To enable imaging of MreB polymers, the MreBs were labeled with Alexa488-maleimide. We have previously shown that Alexa-488 labeling had no significant effect on the functional properties of *Li*-MreB^39^. In 300 mM KCl, 2 mM MgCl_2_ and 0.1 mM CaCl_2_, *Li*-MreB formed large bundled assemblies (Fig. 2A) while *Rr-*MreB formed extensive sheets (Fig. 2C). *Li*-MreB formed similar assemblies in 300 mM NaCl as in 300 mM KCl, indicating that sodium and potassium may have similar effects on MreB assembly (Supp. 2F). *Li*-MreB purified with the PF method under the salt composition determined for *Li* cells (296 mM NaCl, 5 mM KCl, 0.38 mM MgCl_2_, 1 mM CaCl_2_,) formed similar superstructures based on the interlacement of curved ribbon-like sheets (Fig. 2B) as in the presence of 300 mM KCl, 2 mM MgCl_2_, 0.1 mM CaCl_2_ (Fig. 2A) (Supp. Movie 1). The KCl sensitivity of *Rr-*MreB polymerization shows an optimum (Supp. 2C) at 200 mM and formed similar structures (Fig. 2D) as *Li*-MreB in 300 mM KCl (Fig. 2A)*. Li*-MreB and *Ec*-MreB formed similar structures in the presence of 300 mM KCl, 2 mM MgCl_2_, 0.1 mM CaCl_2_ (Fig. 2A and E), which resembled the *Ec*-MreB assemblies purified under the intracellular salt composition (219 mM NaCl, 38 mM KCl, 2.38 mM MgCl_2_, 0.24 mM CaCl_2_) (Fig. 2F)*.*

### Reorganization of MreB polymers by changes in calcium levels

**Figure 3:**
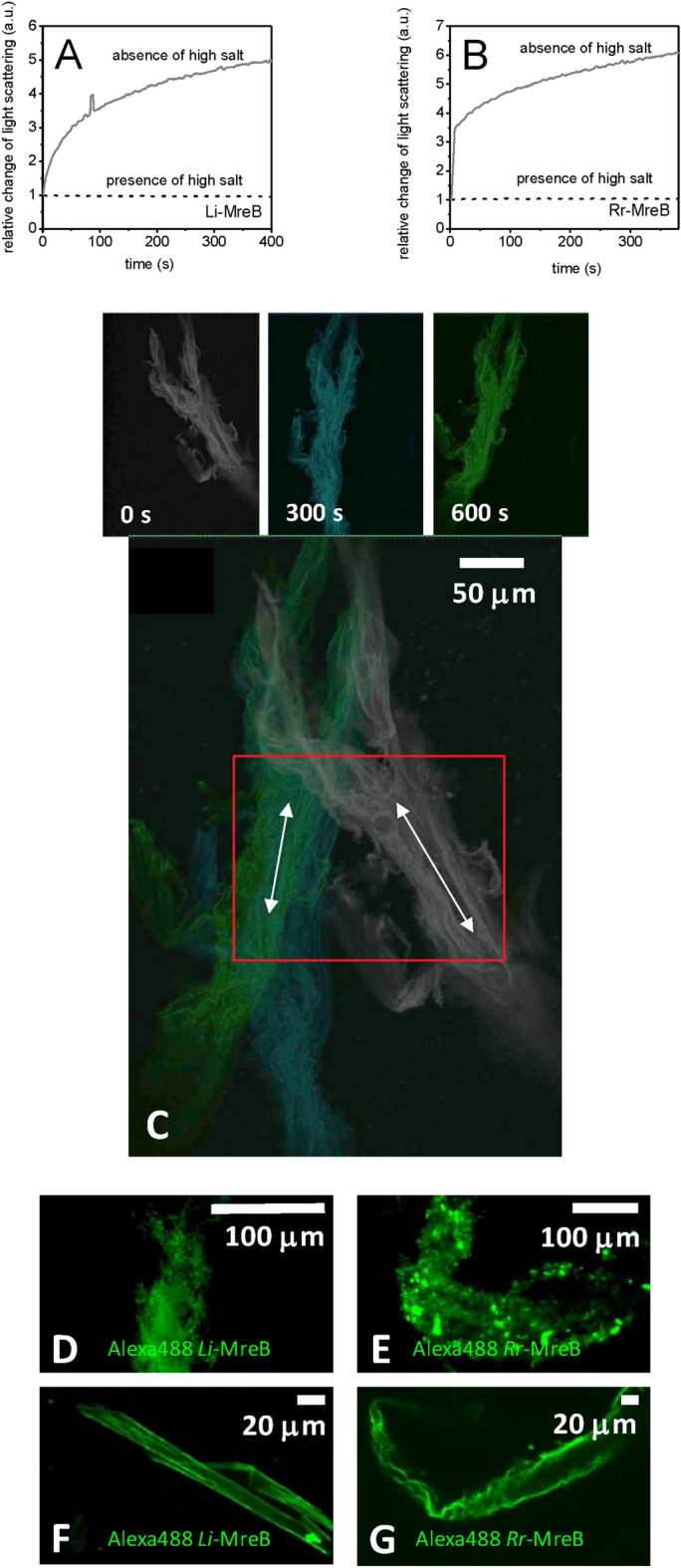
Effect of divalent cations on MreB monomers and polymers. (**A** and **B**) Relative light scattering change of 50 µM (**A**) *Li*-MreB or (**B**) *Rr-*MreB solutions on adding 2 mM calcium to monomers (purified under denaturing conditions) in the absence of high salt (2 mM TRIS.HCl, 0.1 mM CaCl_2_, 0.2 ATP, 1 mM DTT, pH 8.0) (gray line) or to polymers in the presence of high ionic conditions (300 mM KCl, 2 mM MgCl_2_, 0.1 mM CaCl_2_) (dashed line). (**C**) Microscopy time-lapse snapshots from the entropic motion of Alexa488 labeled *Li*-MreB superstructures under high salt conditions (300 mM KCl, 2 mM MgCl_2_, 0.1 mM CaCl_2_). The state of polymer before (gray), 5 min after (cyan) and 10 min after (green) the 2 mM calcium addition. On the overlay image, white arrows indicate that the length change of assemblies contracted by calcium addition (lower panel). (**D** and **E**) Microscopy images of Alexa488-labeled (**D**) *Li*-MreB or (**E**) *Rr-*MreB polymers after 6 mM EDTA treatment, in the absence of Ca^2+^ and Mg^2+^. (**F** and **G**) Microscopy images of Alexa488 labeled (**F**) *Li*-MreB or (**G**) *Rr-*MreB polymers after 6 mM EGTA treatment, in the absence of Ca^2+^ and presence of Mg^2+^.

*Li*-MreB shows monovalent cation dependent sensitivity to calcium ion concentrations in the range of 0.1–2 mM. In the absence of KCl and MgCl_2_, a fivefold increase in light scattering was observed on the addition of 2 mM CaCl_2_ to *Li*-MreB monomers (50 µM). However, in the presence of 300 mM KCl and 2 mM MgCl_2_ the signal from the polymers was unchanged on the calcium addition (dashed line in Fig. 3A). Similar effects were observed for *Rr-*MreB (Fig. 3B). To further investigate these effects, we carried out time-lapse microscopy imaging to explore any structural changes of fluorescently labeled *Li*-MreB polymers in response to calcium treatment. The initial large assemblies (gray, Fig. 3C), appeared to shorten and become less flexible after 5 min (cyan, Fig. 3C) and 10 min (green, Fig. 3C) on the addition of 2 mM CaCl_2_ (Movie 1). White arrows in Fig. 3C indicate the shortening of the MreB assembly, by the contraction of the whole structure. At the start, the superstructure moves relatively freely. After calcium treatment, the thermal motion of the superstructure slowed and stopped. Both characteristics suggest a calcium-induced structural change in the MreB assembly. However, after 6 mM EDTA treatment in 300 mM KCl, in the absence of divalent cations, the *Li*-MreB and *Rr-*MreB polymers precipitated (Fig. 3D and E)*.* The addition of 6 mM EGTA, rather than EDTA, in the absence of calcium ions, led to the *Li*-MreB superstructures remodelling into a single ribbon-like sheet (Fig. 3F) (Supp. Movie 2), while *Rr-*MreB formed irregular bundled assemblies (Fig. 3G). These data show that calcium influences the formation of MreB polymer superstructures.

**Figure 4:**
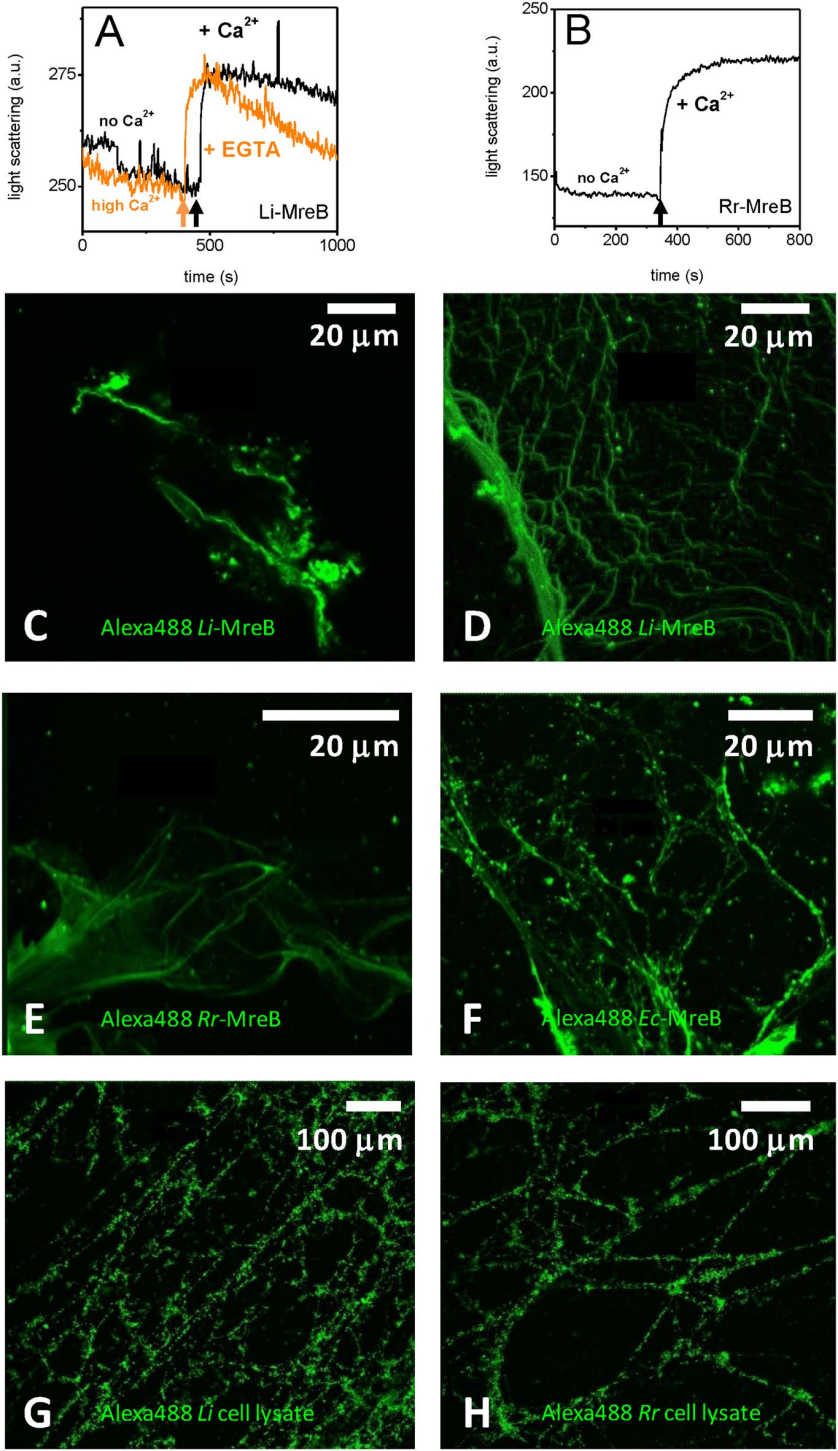
Rapid changes in calcium levels caused structural reorganization of MreB polymers. (**A**) Under high ionic conditions (300 mM KCl, 2 mM MgCl_2_, 0.1 mM CaCl_2_), 6 mM EGTA was added to polymerized *Li*-MreB (50 µM) (black line) then a 2 mM calcium addition caused a jump in light scattering. EGTA and Ca^2+^ were added in the opposite order (orange line). (**B**) Under high ionic conditions (300 mM KCl, 2 mM MgCl_2_, 0.1 mM CaCl_2_), 6 mM EGTA was added to *Rr-*MreB polymers (50 µM) (black line) then 2 mM Ca^2+^ treatment caused a similar response in light scattering as in case of *Li*-MreB (arrows indicate the addition of Ca^2+^ or EGTA). The initial state of MreB at the beginning of the experiment was similar to that shown in Fig. 2. (**C** and **D**) Confocal fluorescent microscope images of Alexa488-labeled *Li*-MreB polymers (**C**) after the consecutive application of 2 mM Ca^2+^ then 6 mM EGTA or (**D**) first 6 mM EGTA then 2 mM Ca^2+^. (**E** and **F**) Images of Alexa488-labeled (**E**) *Rr-*MreB, (**F**) *Ec*-MreB polymers, after consecutive application of 6 mM EGTA then 2 mM Ca^2+^. (**G** and **H**) Microscopy images of (**G**) *Li*-MreB or (**H**) *Rr-*MreB overexpressing *E. coli* cell lysates (diluted two times with distilled water and spread on glass slides) after 6 mM EGTA then 2 mM calcium addition. All cysteines of the sample were labeled with Alexa488-maleimide.

To better understand the role of calcium, we carried out experiments in which the responses of polymers were monitored by light scattering on fast changes in calcium ion concentrations. In the presence of 6 mM EGTA, where free calcium was eliminated from the solution, the addition of 2 mM Ca^2+^ led to a jump in light scattering (black line in Fig. 4A), which subsequently decreased slowly, indicating a moderate size change of the *Li*-MreB (50 µM) polymers. Similarly, after extended incubation with 2 mM CaCl_2_ followed by a sudden switch to EGTA (6 mM) a fast increase in light scattering was observed (orange line in Fig. 4A) followed by a gradual decrease, indicating that the *Li*-MreB polymers went through a relatively quick supramolecular reorganization. Likewise, a sudden change of calcium level, by consecutive application of EGTA then calcium, had a similar effect on *Rr-*MreB sheets indicated by a rapid increase in light scattering (Fig. 4B). To explore how calcium affects MreB polymers in the presence of EGTA, we followed the change with fluorescence microscopy. Alexa488 labeled *Li*-MreB assemblies in the presence of 2 mM CaCl_2_ were dissociated and precipitated after the addition of 6 mM EGTA (Fig. 4C), explaining the quick change of light scattering after this treatment (Fig. 4A, orange line). Surprisingly, in the presence of 6 mM EGTA, *Li*-MreB polymers formed novel web-like structures after 2 mM CaCl_2_ addition (Fig. 4D) as was also the case for *Rr-*MreB (Fig. 4E) and *Ec*-MreB (Fig. 4F) polymers. Possibly, magnesium can replace calcium in binding to the MreB polymers to stabilize the assemblies, since the elimination of calcium by EGTA in the presence of magnesium did not affect the MreB assemblies. Subsequent addition of calcium, in excess of the local EGTA concentration, reorganized the MreB filaments. Microscopy images of whole cell lysate from *E. coli* overexpressing *Li*-MreB or *Rr-*MreB (Fig. 4G and H, respectively), in which all cysteines of the entire sample were labeled with Alexa488-maleimide, showed extensive web-like structures after treatment with EGTA (6 mM) followed by CaCl_2_ (2 mM excess). This indicates that changes in intracellular calcium levels may play a role in intracellular polymer reorganization.

### The persistence length of MreB under various salt conditions

**Figure 5:**
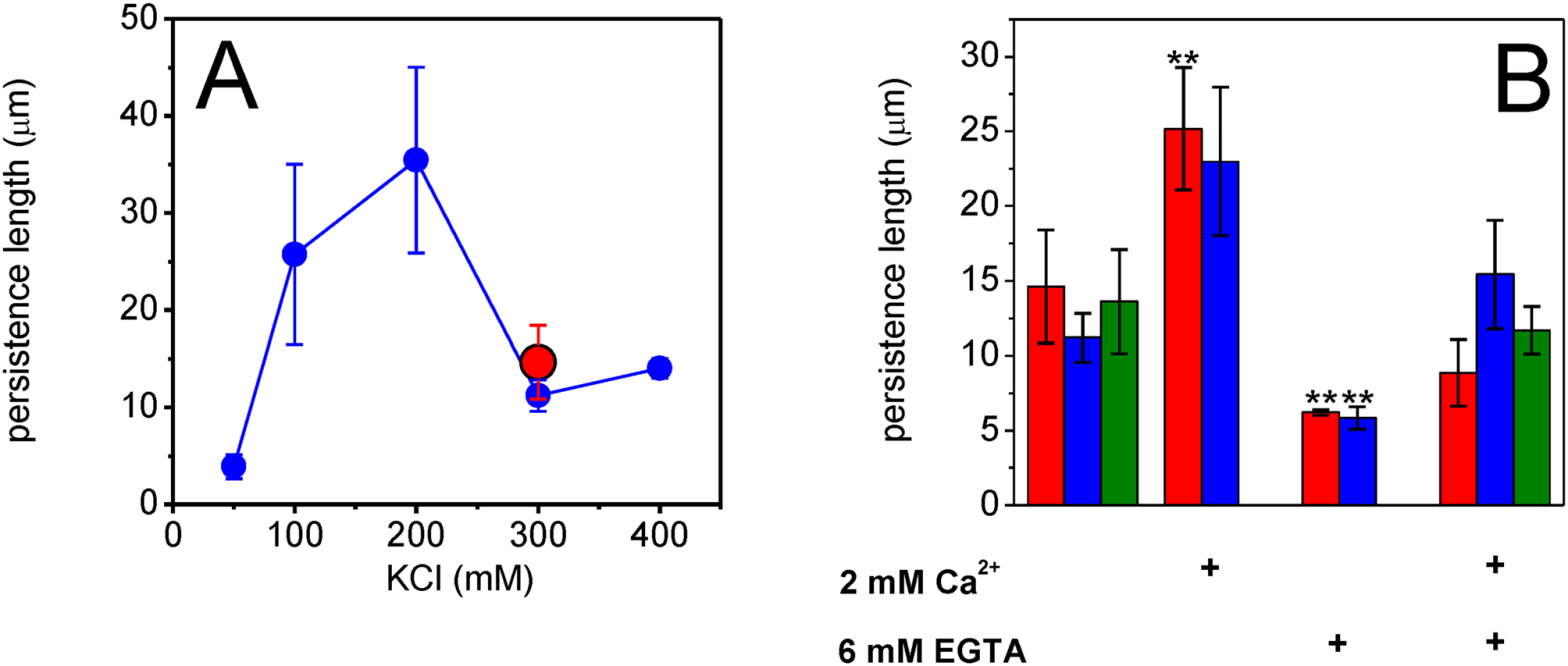
Persistence lengths of different MreB assemblies. (**A**) Analysis of microscopy images fitting with the WLC model in the Easyworm software. Alexa488-labeled *Rr-*MreB polymers were grown under different KCl conditions (50–400 mM), which reveals the longest persistence length at its polymerization optimum at 200 mM of KCl (blue circles). The persistence length of Alexa488 labeled *Li*-MreB is shorter at its polymerization optimum at 300 mM KCl (red circle). (**B**) The persistence length of Alexa488-labeled *Li*-MreB assemblies (red columns) changed significantly in the presence of 2 mM calcium, and in the absence of calcium by the treatment with 6 mM EGTA, and *Rr-*MreB assemblies (blue columns) showed a non-significant change in the presence of 2 mM calcium, but a significant change in the presence of 6 mM EGTA. Both assemblies show non-significant differences to the pretreated samples on subsequent addition of 2 mM calcium after EGTA treatment. The persistence length of Alexa488-labeled *Ec*-MreB assemblies (green columns) did not show a large difference on the addition of 6 mM EGTA followed by 2 mM calcium. Error bars refer to mean ± SD of five independent measurements. Double asterisks indicate statistically significant difference relative to the initial conditions. The analysis was based on a two-sample *t*-test, *p* < 0.005.

Since ionic conditions have substantial effects on MreB polymerization and superstructure reorganization, we sought to characterize the flexibility of MreB polymers under various salt conditions. We analyzed the microscope images with Easyworm software to estimate the persistence lengths of the MreB structures. Ionic strength was effective in changing the polymerization properties of *Rr-*MreB (Supp. 2B, C, D), therefore the persistence length was examined as the function of the KCl concentration (Fig. 5A). The buffers contained different amounts of KCl (50–300 mM) and 2 mM MgCl_2_, 0.1 mM CaCl_2_. Persistence lengths were ionic strength dependent and fell in the range of 3.9–35.5 µm (images not shown). Interestingly, the optimal polymerization conditions of *Rr-*MreB (200 mM KCl) resulted the longest persistence length and the lowest flexibility of polymers. However, the persistence lengths of *Li*-MreB and *Rr-*MreB polymers in the presence of 300 mM KCl were similar (14.6 µm), indicating similar flexibilities. For reference, in the case of actin, the persistence length is 12 µm in the activated state of the thin filaments, in the presence of calcium and binding of the tropomyosin-troponin complex^40^. The persistence length of ADP-bound state of the actin filaments is 9 ± 0.5 µm, while the filaments become much stiffer in the absence of calcium (20 ± 1 µm). The persistence lengths estimated here for MreB superstructures varied within a broad range (3.9–35.5 µm), which is similar to the range observed for actin filaments. Interestingly, in the presence of 100 mM KCl the persistence length of *Rr-*MreB (25.73 ± 9.3 µm) polymers was similar to the values of actin thin filaments in the absence of calcium (20 ± 1 µm)^40^.

These data indicate a potential role of calcium in remodeling of MreB superstructures. In the presence of 300 mM KCl, 2 mM MgCl_2_, 0.1 mM CaCl_2_ the persistence length of *Li*-MreB, *Rr-*MreB and *Ec*-MreB polymers were almost identical (Fig. 5B) around 13 µm. On addition of 2 mM CaCl_2_, the persistence lengths of *Li*-MreB and *Rr-*MreB polymers increased to 23–25 µm, the filaments stiffened. In the absence of calcium (6 mM EGTA), both MreBs were more flexible (Lp ~ 6 µm), and subsequent calcium addition (2 mM) caused reorganization of MreBs resulting in slightly stiffer structures and significantly different persistence length *Li*-MreB (Lp = 8.9 µm), *Rr-*MreB (Lp = 15.4 µm) and *Ec*-MreB (Lp = 11.69 µm).

## Discussion

Here, we used two methods, ion-selective electrodes/colorimetry and flame photometry, to measure the concentrations of Na^+^, K^+^ and Ca^2+^ cell extracts, and used ion-selective electrodes/colorimetry to determine the Mg^2+^ and Cl^−^ concentrations. These methods reveal the average intracellular ion conditions and do not report inhomogeneous distributions in different areas of a cell. Nevertheless, the agreement between the Na^+^, K^+^ and Ca^2+^ levels, measured by the two techniques, suggests that these average values are accurate, and that in general the levels of sodium ions exceed potassium ions in bacteria. Bacterial cells can exchange monovalent cations with the media to adjust intracellular ionic strength during osmoadaptation^41^. The types of broth used here in growing bacterial cultures were major source of sodium ions. The conditions for culturing *Leptospira interrogans* (Korthof), *Escherichia coli* (Luria–Bertani) and *Bacillus subtilis* (Mueller–Hinton) contain 54, 85 and 342 mM sodium ions, respectively, which show a different relationship to the measured intracellular sodium ion concentrations, *Leptospira interrogans* (296 mM), *Escherichia coli* (219 mM) and *Bacillus subtilis* (122 mM). We measured almost identical intracellular ion concentration-derived ionic strengths in *Leptospira interrogans* (235 mM) and *Escherichia coli* (237 mM)*,* which mainly resulted from sodium and chloride ions. The intracellular ionic strength in *Bacillus subtilis* was significantly lower (130 mM), suggesting that non-ion osmolytes may have a significant role in maintaining the osmotic balance.

**Figure 6:**
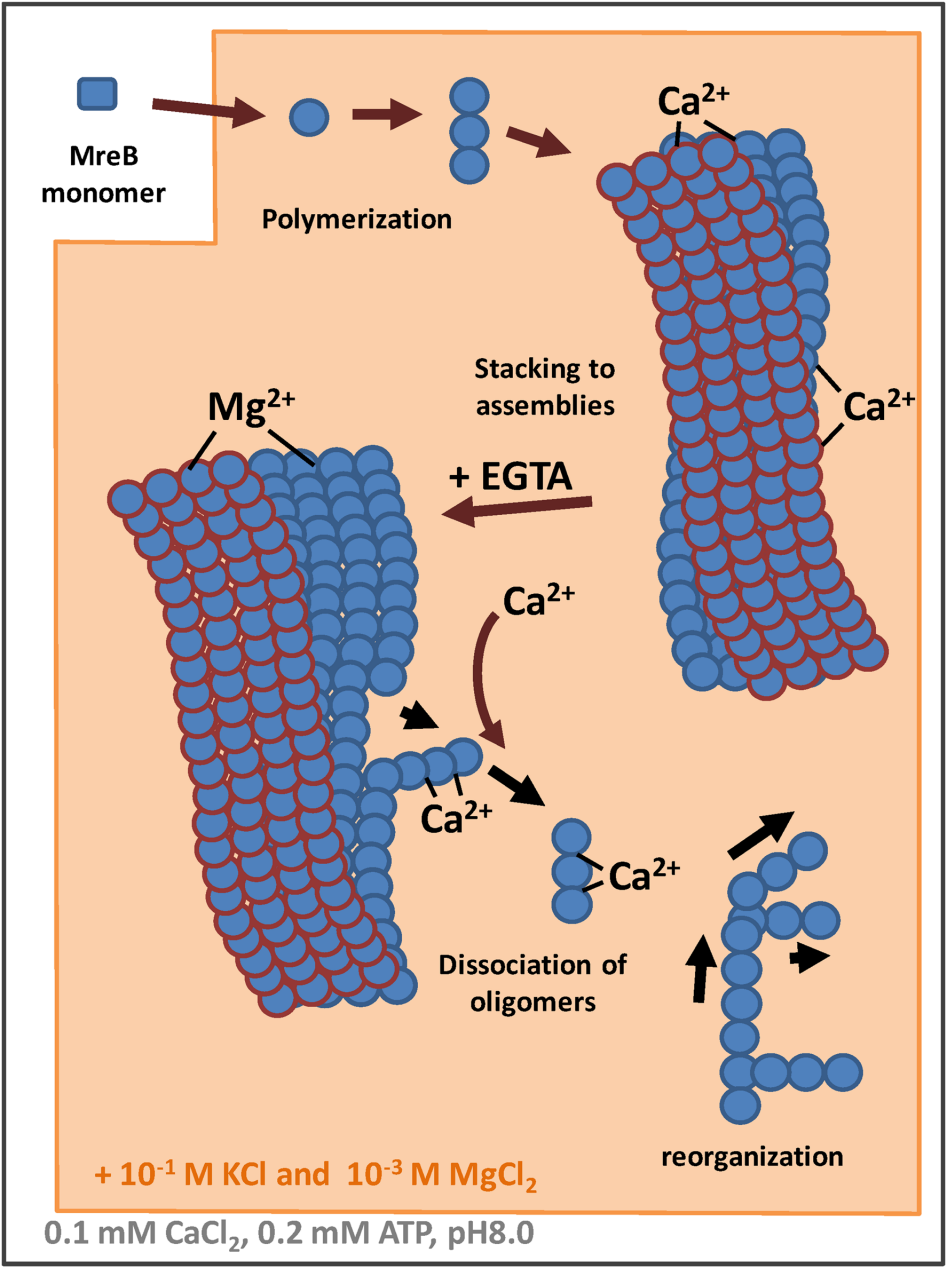
A hypothetical model to interpret the dynamics of MreB polymers.

The in vitro polymerization and formation of MreB superstructures is highly dependent on the presence of cations. Millimolar magnesium and hundred millimolars potassium or sodium are necessary for efficient MreB polymerization. Addition of calcium caused the stacking into ribbon-like structures and large assemblies, and we hypothesize that calcium binding may change the strain in filaments. Subsequent calcium depletion, via EGTA treatment, reordered the polymers into extensive sheets in the presence of magnesium, and further treatment with calcium led to fissured monolayer sheets and the dissociation of filaments into web-like structures (Fig. 6). Structural studies have shown that pairs of MreB protofilaments associate together in an antiparallel manner, while molecular dynamics simulations suggest the possibility of curvature in the protofilaments^42,43^. The in vitro assemblies observed here are likely to be formed by the non-polarized filaments associating side by side. In vivo the assemblies will also be stabilized by membrane binding^43^. Molecular dynamics simulations have shown that the twist of MreB double protofilaments can be reduced by membrane binding. The moderated dynamics of MreB filaments resulted in shorter filaments, and possibly provides tuning to their flexibilty and length^44^. The untwisted antiparallel structure of pairs of protofilaments can allow bending of the MreB filaments, which may stabilize the curvature of a membrane^44^. High calcium concentrations^45^, near a membrane, may induce longer persistence lengths in MreB polymers. Thus, we hypothesize that local calcium concentration changes may lead to the reshaping of membranes. Through calcium-ion induced stiffness, MreB filaments may contribute to specifying membrane curvature at points of membrane-MreB interaction (Supp. 4). Presumably, the final cell shape will be a product of the MreB properties and the ionic milieu. Calcium concentration-induced changes of MreB structure may also participate in membrane remodelling during cell division or osmotic adaptation. Rr-MreB polymerization is more sensitive to monovalent cations than Li-MreB, which suggests that the shape and stability of cytoskeletal systems will vary between organisms under similar intracellular conditions. Since, the high salt conditions present in bacteria fluctuate in response to osmotic shock, the influence on the stability of MreB scaffold and its ability to reattach bacterial membranes to cell wall is likely to be affected by divalent cations. Varying ionic conditions do not in general change cell shape, however they are known to modify membrane stiffness and enzyme activity, which effects the stability of bacterial envelope^46^. However, the effect of calcium levels appears to be more sensitive. It is likely that calcium fluxes may have a role in regulating and remodelling the in vivo MreB cytoskeleton, which in turn may influence the mobility and localization of links between the membrane and cell wall during the cell division.

One of the limitations of our study is that due to the limited number of species analyzed, it is not clear whether the differences seen are species specific or common features of several relative strains. But we highlighted the relevance of intracellular ion conditions influence on MreB polymer formation.

## Methods

### Determination of intracellular ion concentrations

The *Escherichia coli*, *Bacillus subtilis* and *Leptospira interrogans* cells (gifts from the Department of Medical Microbiology and Immunology) were grown until OD_600nm_ = 1 in suitable media. Luria–Bertani broth for *E. coli*: 10 g/L tryptone, 5 g/L yeast extract, 5 g/L NaCl; Mueller–Hinton broth for *B. subtilis*: 2 g/L meat infusion, 17.5 g/L casein hydrolysate, 1.5 g/L starch; Korthof broth for *L. interrogans*: 1.4 g/L NaCl, 0.88 g/L Na_2_HPO_4_, 0.8 g/L peptone, 0.24 g/L KH_2_PO_4_, 0.04 g/L CaCl_2_, 0.04 g/L KCl, 0.02 g/L NaHCO_3_, added rabbit serum and rabbit hemoglobin (sterile 8 mL inactivated blood serum and 0.8 mL sterile haemoglobin solution were added in 100 mL broth).

Cells were harvested by centrifugation (4500 × *g* for 10 min), then the extracellular medium was removed by washing. Five times the pellet volume of distilled water was added, incubated for 2 min at RT, and subsequently inverted gently ten times and centrifuged again (4500 × *g* for 10 min). The pellet volume was measured in a scaled 2 mL Eppendorf tube. Cells killed and the extracellular water was evaporated by heat treatment (100 °C for 20 min). The cell pellets were resuspended in five times the pellet volume of water, frozen and subsequently boiled to generate a homogenous cell lysate. The slurry was centrifuged for 10 min at 100,000 × *g*. Ion concentrations were measured from the supernatants. Unbound Na^+^, K^+^, Cl^−^ (ion-selective electrode) Ca^2+^ and Mg^2+^ (colorimetry) levels were analyzed using a COBAS INTEGRA 400 plus analyzer (Roche Diagnostics, GmbH, Mannheim, Germany) following the manufacturer's instructions. Total (protein-bound and free) Na^+^, K^+^ and Ca^2+^ were also measured by flame photometry (Efox 5053/Eppendorf). The intracellular ion concentrations are equal to five times of measured values. The final ionic strength (IS) was derived using the following equation:

1 $$\left[ {{\text{IS}}} \right] = \frac{1}{2}\mathop \sum \limits_{{{\text{i}} = 1}}^{{\text{n}}} {\text{c}}_{{\text{i}}} {\text{z}}_{{\text{i}}}$$

where *c*_*i*_ and *z*_*i*_ are the molar concentrations and charge of the ions, respectively.

### Expression of MreB

The *Leptospira interrogans* MreB gene (gene bank accession number: AAS69864.1), the *Rickettsia rickettsii* MreB gene (gene bank accession number: ABY73136.1) and the *Escherichia coli* MreB gene (gene bank accession number: AJF45056.1) sequences were codon optimized for expression in *E. coli*, synthesized (GenScript) and engineered into the pSY5 plasmid which encodes an 8-histidine tag, followed by a PreScission protease (GE Healthcare Life Sciences) cleavage site prior to the protein sequence. The construct was verified by DNA sequencing. The DNA construct encoding MreB was transformed into *E. coli* BL21 DE3 pLysS strain (Novagen). Cell cultures were grown in Luria Broth medium at 37 °C supplemented with ampicillin until reaching OD600 = 0.6. Protein expression was induced by adding IPTG to a final concentration of 1 mM followed by incubation overnight at 20 °C. Cells were harvested by centrifugation and pellets were stored at − 20 °C^39^.

### Purification of MreB

We used a novel method for the isolation of MreB polymers, which we refer to as the polymer filtration (PF) method.* E. coli* cell pellets were resuspended in a buffer consisting of 2 mM TRIS.HCl, 50–300 mM KCl, 2 mM MgCl_2_, 0.1 mM CaCl_2_, 0.2 mM ATP, 1 mM DTT (pH 8.0). The slurry was treated with DNase (0.01 U/mL) (PanReac, AppliChem) and lysozyme (0.01 mg/mL) (Sigma-Aldrich) overnight at 4 °C. MreB polymers (Supp. 3C) were filtered first on a paper filter and then washed and filtered again on a 0.45 µm membrane filter. The purity of MreB stocks were assessed with SDS-PAGE (Supp. 1) (Supp. 1C was cropped from Supp. 3D).

For the comparison of MreB polymers prepared by the PF method, we also purified MreB monomers on a Ni–NTA column under denaturating conditions with minor changes of protocol what we published before^39^. A cell pellet was resuspended and homogenized in a denaturing lysis buffer (6 M guanidine-HCl, 0.1 M NaH_2_PO_4_, 10 mM TRIS.HCl, pH 8.0). After centrifugation (30,000 × *g* at 4 °C for 2 h) the supernatant was loaded onto a Ni–NTA column and incubated overnight at 4 °C. Then the column was washed with a buffer containing 8 M urea, 0.1 M NaH_2_PO_4_, 10 mM TRIS.HCl. A descending pH gradient from pH 8.0 to 4.0 was applied to elute the protein. MreB containing fractions were pooled and dialyzed overnight against a buffer containing 2 mM TRIS.HCl, 0.1 mM CaCl_2_, 0.2 mM ATP, 1 mM DTT, pH 8.0) in order to refold the MreB monomers. The renatured MreB were clarified by ultracentrifugation (100,000 × *g*, 4 °C, 30 min). The MreB stocks were either used immediately or flash-frozen with liquid nitrogen in small volumes. Concentrations of MreBs were determined by Bradford Assay (Bio-Rad) using a Jasco v-660 photometer (Jasco Corporation).

### Fluorescent labeling of MreB

The cysteines of MreBs were labeled with Alexa Fluor 488 C5 maleimide or with Alexa Fluor 568 C5 maleimide. Prior to initiating of the labeling reaction, DTT was removed from the solution by overnight dialysis. Next, MreB polymers were incubated in the presence of fivefold molar excess of the fluorophore for 1 h on ice. The excess fluorophore was removed by overnight dialysis. However, labeled MreB polymers were not but monomers were clarified by ultracentrifugation (100,000 × *g*, at 4 °C for 30 min) and the concentrations of protein and fluorophore in the supernatant were determined using spectrophotometry (ε_Alexa488_ = 73,000 M^−1^ cm^−1^, ε_Alexa568_ = 88,000 M^−1^ cm^−1^, ε_LiMreB_ = 10,555 M^−1^ cm^−1^, ε_RrMreB_ = 15,025 M^−1^ cm^−1^, ε_EcMreB_ = 7575 M^−1^ cm^−1^). The ratio of labeling was calculated as the concentration ratio of the probe to the protein and was found to be approximately 0.1 in cases of MreB polymers and more than 0.5 in case of monomers.

### In vitro polymerization and polymer remodelling assays of MreB

The polymerization kinetics of MreB was investigated using light scattering assays with a Perkin Elmer LS-50 spectrofluorimeter. The excitation and emission monochromators were set to wavelength 400 nm and the excitation and emission slits to 2.5 nm. The samples (2 mL) were stirred continuously with a magnetic stirrer during the polymerization process. Polymerization was initiated by adding monovalent and/or divalent cations, as indicated. The same set up was used to study the time dependent and ionic concentration dependent morphology changes of MreB polymers. The settings for the method based on our protocol and experience^39^.

### Fluorescence microscopy

In these experiments 15 μL of fluorescent labeled MreB was dropped on slides, incubated in the presence of a buffer containing 300 mM KCl, 2 mM MgCl_2_, 0.1 mM CaCl_2_, unless otherwise indicated, then covered by coverslips. The morphologies of the labeled MreB assemblies were analyzed using a Leica TCS SP confocal scanning microscope system (Leica Microsystems GmbH Germany) equipped with a 10-63X objective lens. Image acquisition was carried out a fluorescent-probe specific wavelengths, Alexa488 (ex.: 488 nm, em.: 515–560 nm) and with Alexa568 (ex.: 543 nm, em.: 600–700 nm). The typical vertical stacking height of images was 1–3 µm.

### Determination of persistence length of MreB assemblies

To determine the persistence length of the filaments we used *Easyworm*, an open-source software tool used to determine the mechanical properties of protein chains (Lamour et al.; licensee BioMed Central Ltd. 2014), coded in MATLAB^47^. Representative confocal microscopy images were selected and fitted with the worm-like chain (WLC) model. Resultant persistence lengths (Lp) were calculated from the function of End2End length versus contour length of assemblies.

### Data analysis

Data are presented as means ± standard deviations (SD) throughout. Comparisons were performed using Students *T*-test and statistically significant differences between groups were defined as *p* values < 0.05 or < 0.005 and are indicated in the legends of figures.

## Supplementary information

Supplementary Video 1.

Supplementary Information.

Supplementary Video 2.

Supplementary Video 3.

## Data Availability

The datasets generated during and/or analysed during the current study are available from the corresponding author on reasonable request.
