# Author Correction: Intracellular ion concentrations and cation-dependent remodelling of bacterial MreB assemblies

**DOI:** 10.1038/s41598-020-74830-2

**Published:** 2020-10-20

**Authors:** Dávid Szatmári, Péter Sárkány, Béla Kocsis, Tamás Nagy, Attila Miseta, Szilvia Barkó, Beáta Longauer, Robert C. Robinson, Miklós Nyitrai

**Affiliations:** 1 Department of Biophysics, Medical School, University of Pécs, Pécs, Hungary; 2 MTA-PTE Nuclear-Mitochondrial Interactions Research Group, Pécs, Hungary; 3 Department of Medical Microbiology and Immunology, Medical School, University of Pécs, Pécs, Hungary; 4 Department of Laboratory Medicine, Medical School, University of Pécs, Pécs, Hungary; 5 Szentágothai Research Center, University of Pécs, Pécs, Hungary; 6 School of Biomolecular Science and Engineering (BSE), Vidyasirimedhi Institute of Science and Technology (VISTEC), Rayong, Thailand; 7 Research Institute for Interdisciplinary Science (RIIS), University of Okayama, Okayama, Japan

Correction to: * Scientific Reports* 10.1038/s41598-020-68960-w, published online 20 July 2020

This Article contains a typographical error in Equation 1.

$$\left[\mathrm{IS}\right]=\frac{1}{2}\sum_{\mathrm{i}=1}^{\mathrm{n}}{\mathrm{c}}_{\mathrm{i}}{\mathrm{z}}_{\mathrm{i}}$$

should read:

$$\left[\mathrm{IS}\right]=\frac{1}{2}\sum_{\mathrm{i}=1}^{\mathrm{n}}{\mathrm{c}}_{\mathrm{i}}{\mathrm{z}}_{\mathrm{i}}^{2}$$

