# Supplementary material for: Intracellular ion concentrations and cation-dependent remodelling of bacterial MreB assemblies
Source: Sci Rep. 2020 Jul 20;10:12002. doi: 10.1038/s41598-020-68960-w (PMC7371711; doi:10.1038/s41598-020-68960-w)
Supplement: Supplementary file 2 — Supplementary Information. [file 41598_2020_68960_MOESM2_ESM.pdf]

SUPPLEMENTS

INTRACELLULAR ION CONCENTRATIONS AND CATION-DEPENDENT REMODELLING OF BACTERIAL MREB ASSEMBLIES

Dávid Szatmári[a], Péter Sárkány[a], Béla Kocsis[c], Tamás Nagy[d], Attila Miseta[d,e,], Szilvia Barkó[a], Beáta Longauer[a], Robert C. Robinson[f,g,] and Miklós Nyitrai[a,b,e,*]

[a]*University of Pécs, Medical School, Department of Biophysics, Hungary.*

[b]*MTA-PTE Nuclear-Mitochondrial Interactions Research Group, Hungary.*

[c]*University of Pécs, Medical School, Department of Medical Microbiology and Immunology, Hungary.*

[d]*University of Pécs, Medical School, Department of Laboratory Medicine, Hungary.*

[e]*University of Pécs, Szentágothai Research Center, Hungary.*

[f] *School of Biomolecular Science and Engineering (BSE), Vidyasirimedhi Institute of Science and Technology (VISTEC), Thailand.*

[g]*Research Institute for Interdisciplinary Science (RIIS), University of Okayama, Japan.*

Keywords: *prokaryotic cell, ionic strength, protein conformations, supramolecular assembly, cytoskeleton, bacteria, fluorescence, spectroscopy.*

*Corresponding author

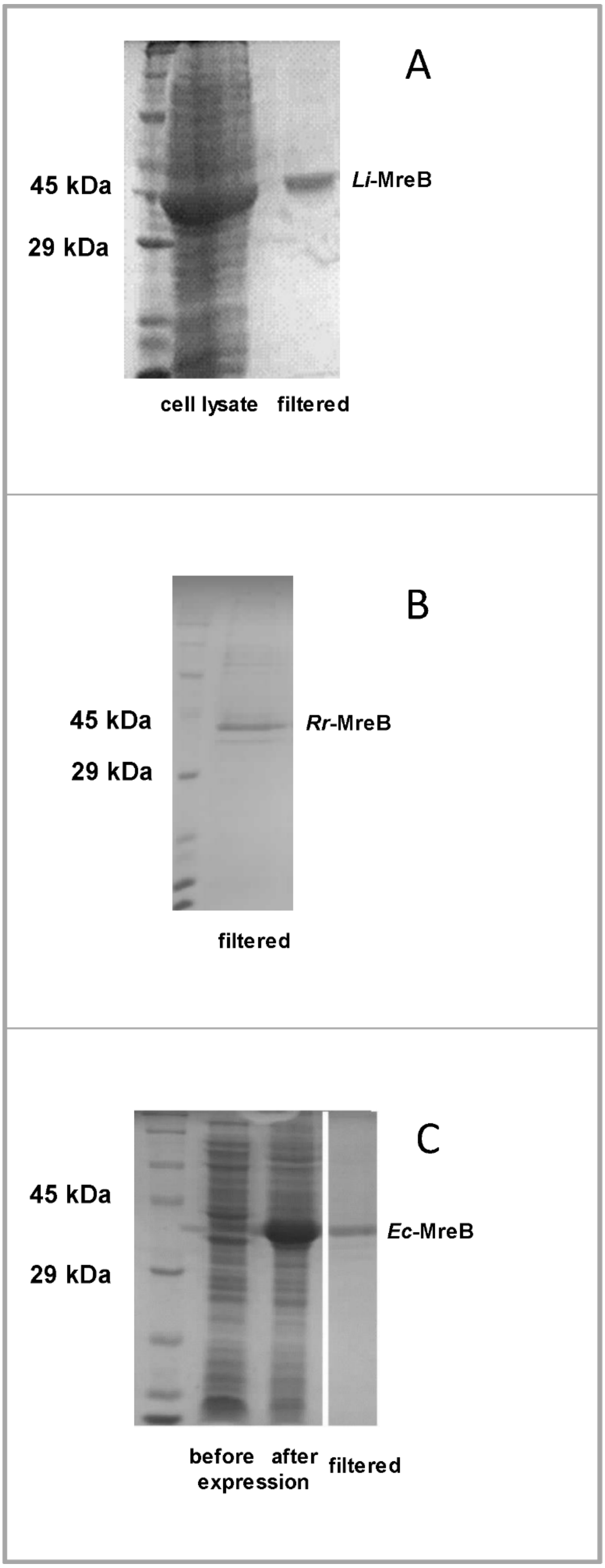
A
45 kDa
29 kDa
Li-MreB
cell lysate
filtered
B
45 kDa
29 kDa
Rr-MreB
filtered
C
45 kDa
29 kDa
Ec-MreB
before
after
expression
filtered

Supplement 1. ***SDS-PAGE gels of MreB filaments prepared by the PF protocol.*** (**A**) MreB from *Leptospira interrogans;* (**B**) MreB from *Rickettsia rickettsii;* (**C**) MreB from *Escherichia coli* (including a cropped image from *Supp.3D*).

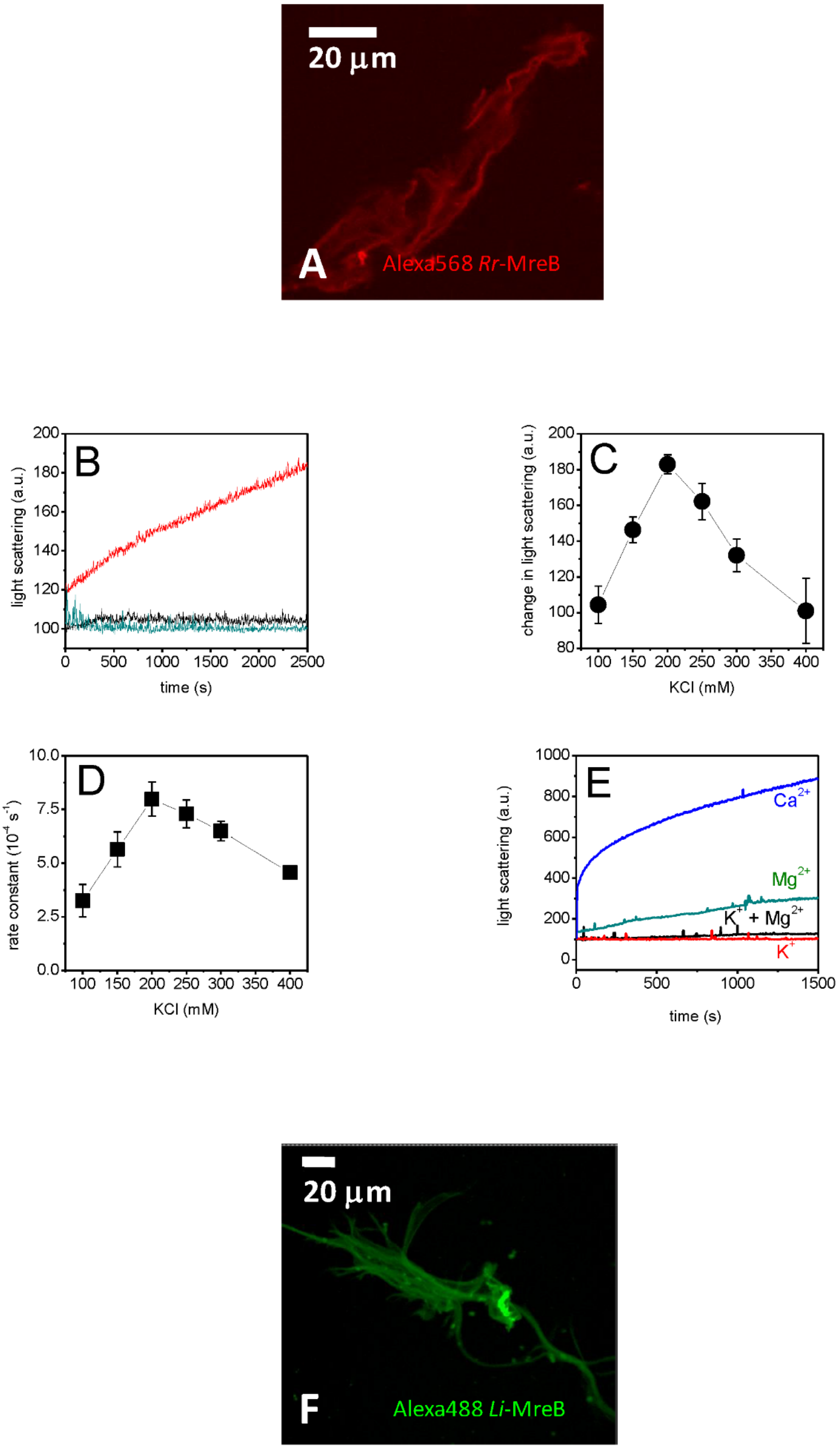
20 μm
A
Alexa568 Rr-MreB
B
light scattering (a.u.)
time (s)
C
change in light scattering (a.u.)
KCl (mM)
D
rate constant (10^-4 s^-1)
KCl (mM)
E
light scattering (a.u.)
time (s)
Ca2+
Mg2+
K+ + Mg2+
K+
20 μm
F
Alexa488 Li-MreB

Supplement 2. (**A**) *Rr*-MreB monomers purified under denaturing conditions, polymerized and labeled with Alexa568-maleimide. (**B**) Light scattering assay following the polymerization of 50 µM *Rr*-MreB initiated by the addition of 2 mM $MgCl_2$ and various concentrations of KCl (black line: 100 mM, red: 200 mM, cyan: 400 mM), in the presence of 0.1 mM $CaCl_2$. (**C**) The relative change in the light scattering signal as the function of the KCl concentration. (**D**) The rates of polymerization obtained by using single exponential fits ($I= s_{max}e^{t/T}$, I is the scattered light intensity, s_{max} is the amplitude, t is elapsed time, T is the rate constant). (**E**) Light scattering change of 50 µM *Rr*-MreB monomers under 100 mM KCl, 2 mM $MgCl_2$ (black line). In 100 mM KCl, in the absence of magnesium (red line). In 4 mM $MgCl_2$, in the absence of potassium (cyan line), and in 4 mM $CaCl_2$ in the absence of potassium and magnesium (blue line). (**F**) Alexa488-*Li*-MreB forms similar purified assemblies in 300 mM NaCl compared to 300 mM KCl (compare with *Fig. 2A*). Error bars refer to mean ±SD of three independent measurements.

A

1: **B.subtilis**	gb|KIX83831.1|	100.00	55.69	56.42	57.44
2: **L.interrogans**	gb|AAS69864.1|	55.69	100.00	55.46	57.69
3: **R.rickettsi**	gb|ABY73136.1|	56.42	55.46	100.00	58.43
4: **E.coli**	gb|AJF45056.1|	57.44	57.69	58.43	100.00

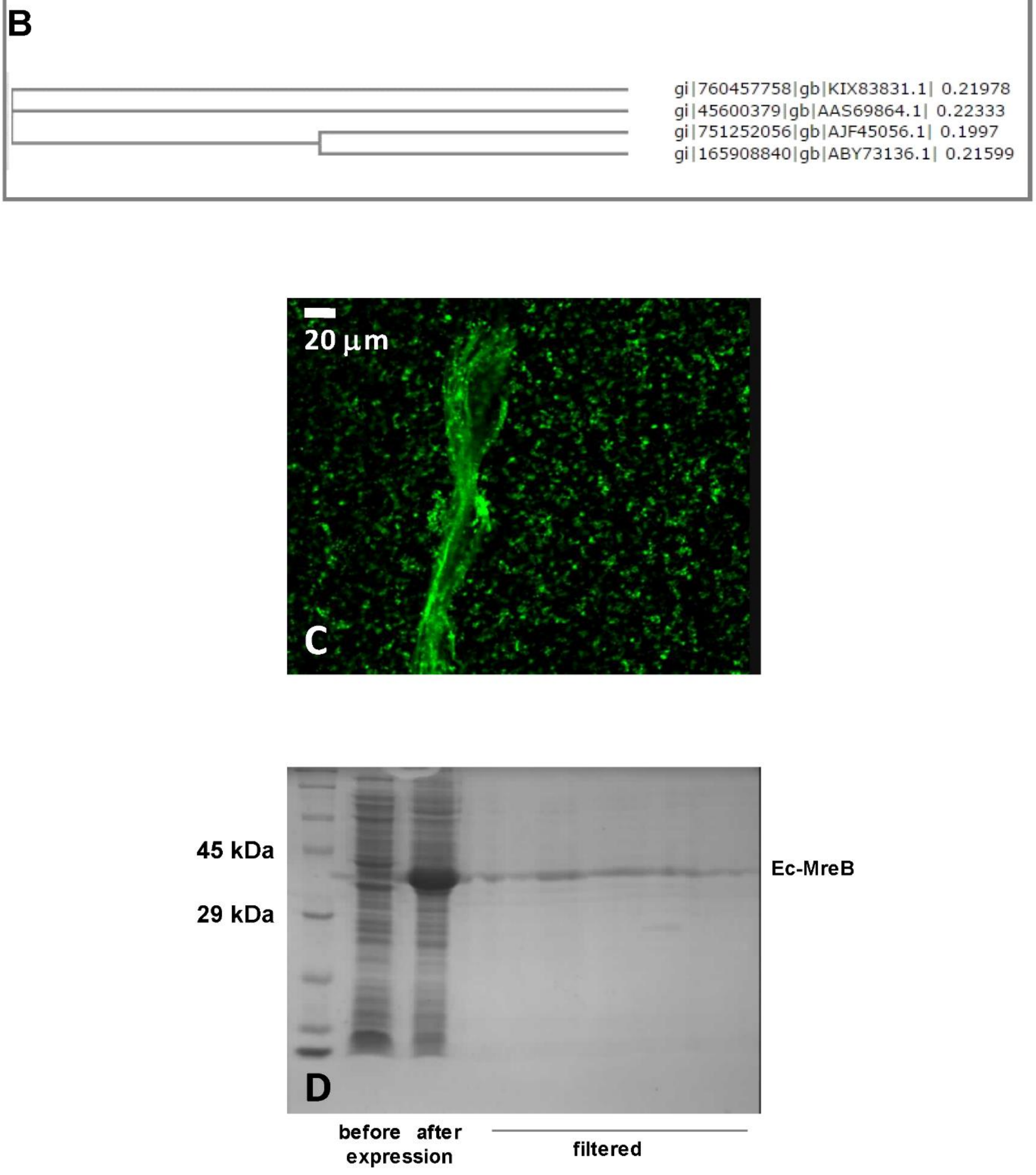

Supplement 3. (**A**) Percent Identity matrix created by Clustal2.1. (**B**) Phylogenetic neighbor-joining tree created by Clustal Omega. (ncbi.nlm.nih.gov) (KIX83831.1:0.227374,(AAS69864.1:0.225,(AJF45056.1:0.219653,ABY73136.1:0.219653):0.00534682):0.00237389) (**C**) *Li*-MreB expressing *E. coli* after lysis in high salt contained buffer (2 mM TRIS.HCl, 300 mM KCl, 2 mM $MgCl_2$, 0.1 mM $CaCl_2$, 0.2 mM ATP, 1 mM DTT, pH 8.0) and DNase I with lysozyme treatment, before filtering. All cysteines in the sample were labeled with Alexa488-maleimide. (**D**) SDS-PAGE of *Ec*-MreB filaments prepared by the PF protocol.

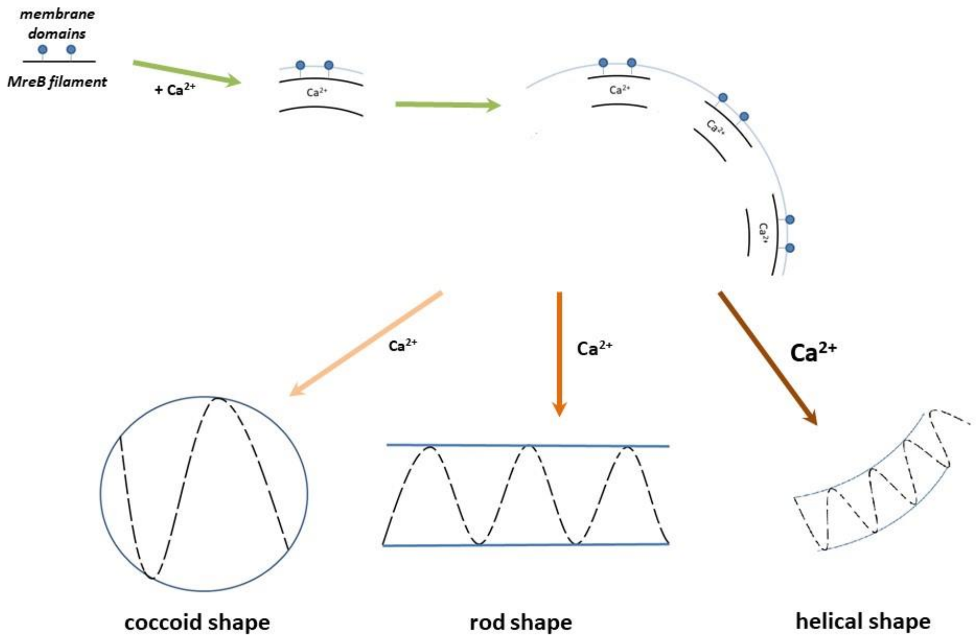

Supplement 4. A hypothetical model to explain how calcium-bound MreB may play a role in cell shape determination.

	E.coli	*L.interrogans*	*B.subtilis*
**[Na^+]/[total cation]**	0.845	0.978	0.808
**[K^+]/[total cation]**	0.145	0.018	0.182
**[Ca^{2+}]/[total cation]**	0.0009	0.003	0.003
**[Mg^{2+}]/[total cation]**	0.009	0.001	0.007
**[Cl^-]/[total cation]**	0.797	0.542	0.7

Table 1. Ratios for each ion as a total of cation concentrations.

Movie 1. The initial large *Li*-MreB assemblies (green), appeared to shorten and become less flexible after ten minutes on the addition of 2 mM $CaCl_2$.

Supplemental Movie 1. Movie generated by ImageJ (NIH/Fiji) from 3D reconstruction of confocal images of *Li*-MreB-Alexa488 assemblies purified by PF method in high salt contained buffer (2 mM TRIS.HCl, 300 mM KCl, 2 mM $MgCl_2$, 0.1 mM $CaCl_2$, 0.2 mM ATP, 1 mM DTT, pH 8.0). Superstructures are based on the interlacement of curved ribbon-like sheets.

Supplemental Movie 2. Movie generated by ImageJ (NIH/Fiji) from 3D reconstruction of confocal images of *Li*-MreB-Alexa488 assemblies purified by PF method in high salt containing buffer (2 mM TRIS.HCl, 300 mM KCl, 2 mM $MgCl_2$, 0.1 mM $CaCl_2$, 0.2 mM ATP, 1 mM DTT, pH 8.0) then treated with 3 mM EGTA. In the absence of calcium, MreB superstructures disassembled and reformed a continuous sheet.
